# Supplementary material for: Far-field acoustic subwavelength imaging and edge detection based on spatial filtering and wave vector conversion
Source: Nat Commun. 2019 Jan 14;10:204. doi: 10.1038/s41467-018-08221-7 (PMC6331623; doi:10.1038/s41467-018-08221-7)
Supplement: Supplementary file 1 — Supplementary Information [file 41467_2018_8221_MOESM1_ESM.pdf]

Supplementary Information for

Far-field acoustic subwavelength imaging and edge detection based on spatial filtering and wave vector conversion

Chu et al.

Supplementary Note 1: Theoretical transmission coefficient and dispersion relation of filter layer

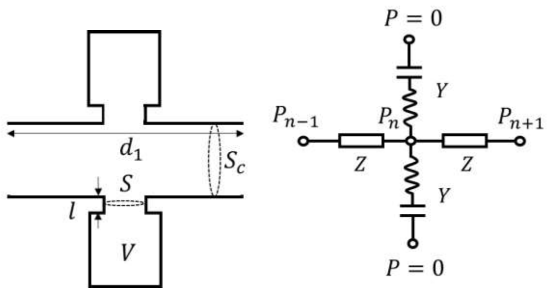

Supplementary Figure 1 Lumped element model of Helmholtz resonator array

When the unit dimension is much smaller than wavelength, the waveguide and the HRs can be represented by the lumped element model, as shown in **Supplementary Figure 1**. Following the method in **[1]**, we have

$$\frac{\partial P}{\partial x} = \frac{ZU}{d_1} \qquad (1)$$

$$\frac{\partial U}{\partial x} = \frac{2YP}{d_1} \qquad (2)$$

In the calculation, P is the averaged excess pressure, U is the volume velocity, $Z = jk_0 d_1 \frac{\rho c}{S_c}$ is the lumped element impedance of the waveguide, and $Y = \frac{-j}{\frac{\omega\rho l}{S} - \frac{\rho c^2}{\omega V}}$ is the admittance of the HR, $k = \frac{\omega}{c}$ is the wave number, $\omega = 2\pi f$ is the angular frequency, and f is the frequency. The system is assumed to be loss-free.

From **Supplementary Equation 1**, and **Supplementary Equation 2**, we get

$$\left(d_1^2 \frac{\partial^2}{\partial x^2} + 2YZ\right) P = 0 \qquad (3)$$

The transmission coefficient of the filter layer is calculated as follows. If the sound source is distributed along the x direction in the waveguide between two HR arrays, **Supplementary Equation 3** will have a source term on the right-hand side and becomes

$$\left(d_1^2 \frac{\partial^2}{\partial x^2} + 2YZ\right) P = s(x), \qquad (4)$$

where $s(x) = s_0 \mathrm{e}^{jk_\mathrm{e}k_0 x}$, and s_0 is a constant indicating the magnitude of the sound source. Then the pressure distribution in the waveguide of the filter layer is

$$P = \frac{s_0}{d_1^2 k_\mathrm{e}^2 k_0^2 + 2YZ} \mathrm{e}^{jk_\mathrm{e}k_0 x}. \qquad (5)$$

The dispersion relation comes from the eigenvalue of **Supplementary Equation 3**. It follows the equation

$$d_1^2 k_x^2 + 2YZ = 0 \qquad (6)$$

Inserting the expressions of Y and Z to **Supplementary Equation 6**, we get

$$k_x^2 = -\frac{2\omega}{d_1 S_\mathrm{c}\left(\frac{\omega l}{S} - \frac{c^2}{\omega V}\right)} \qquad (7)$$

where $c = 343$ m/s, $d_1 = 2.75$ mm, and $S_\mathrm{c} = W_1 h$ is the cross-sectional area of the waveguide perpendicular to x direction. W_1 is the size of the waveguide between the two HR arrays in the z direction. $h = 5$ mm, $l = 0.75$ mm, $S = a_2^2$ is the area of the HR neck tube, where $a_2 = 2.5$ mm. $V = a_1 b_1 c$ is the volume of the HR cavity. The transmission coefficients and dispersion relations of filter1, filter2, and filter 3 are obtained by setting 1) $a_1 = 4.5$ mm, 2) $a_1 = 6$ mm, and 3) $a_1 = 6.5$ mm, respectively.

Supplementary Note 2: Theoretical transmission coefficient of the grating layer T_g^t, T_g^r,

According to reference **[2]**, the curved tube waveguide with wave path length of $2L$ is equivalent to a straight tube of length L with refractive index $n_2 = 2$ if the width of the tube w_1 is much smaller than wavelength, as shown in **Supplementary Figure 2**. The refractive index of the straight tube of length L is n_1=1. Plane wave expansion method **[3]** is used to calculate the theoretical transmission coefficients of different diffraction orders.

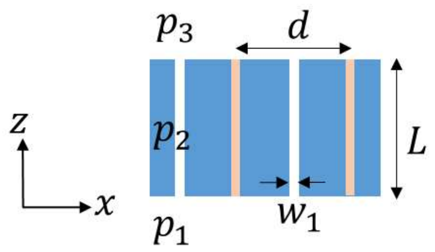

Supplementary Figure 2 Model of the grating layer. Refractive index in white tube is $n_1 = 1$ and refractive index in yellow tube is $n_2 = 2$

The pressure field $p_{\mathrm{in}} = \mathrm{e}^{-jk_{x_0}x}$ incidents onto the grating from bottom. So the pressure field at the bottom area of the grating is $p_1 = e^{j(-k_{x_0}x - k_{z_0}z)} + \sum_m R_m e^{j(-k_{x_m}x + k_{z_m}z)}$. The pressure field at the top area of the grating is $p_3 = \sum_m T_m e^{j(-k_{x_m}x - k_{z_m}(z-h))}$, where $k_{x_m} = k_{x_0} + \frac{2\pi}{d}m$, $k_{x_m}^2 + k_{z_m}^2 = k_0^2$. Since the width w_1 is much smaller than the wavelength, we assume that only the fundamental mode exists in the tubes. Thus the pressure distribution in the tube with refractive index $n_1 = 1$ is $p_{21} = A_1\mathrm{e}^{-jn_1k_0z} + B_1\mathrm{e}^{jn_1k_0z}$, and the pressure distribution in the tube with refractive index $n_2 = 2$ is $p_{22} = A_2\mathrm{e}^{-jn_2k_0z} + B_2\mathrm{e}^{jn_2k_0z}$. Let $v_1 = A_1 - B_1$, $v_1' = A_1\mathrm{e}^{-jn_1k_0L} - B_1\mathrm{e}^{jn_1k_0L}$, $v_2 = A_2 - B_2$, $v_2' = A_2\mathrm{e}^{-jn_2k_0L} - B_2\mathrm{e}^{jn_2k_0L}$. By matching the pressures and z direction velocities at the top ($z = L$) and the bottom ($z = 0$) surfaces of the grating, we obtain the expressions for $v_1, v_1', v_2, v_2{}'$ as follows:

$$-\Gamma_1 v_1 + [H + \Lambda_1]v_1' + 2G^+v_2' = 0$$

$$G^-v_1' - \Gamma_2 v_2 + [2H + \Lambda_2]v_2' = 0$$

$$[H + \Lambda_1]v_1 + 2G^+v_2 - \Gamma_1 v_1' = 2\mathrm{sinc}\left(\frac{k_{x_0}}{2}w_1\right)$$

$$G^-v_1 + [2H + \Lambda_2]v_2 - \Gamma_2 v_2' = 2\mathrm{sinc}\left(\frac{k_{x_0}}{2}w_1\right)\mathrm{e}^{jk_{x_0}(-\frac{d}{2})}$$

where $\Gamma_1 = \frac{1}{j\sin(k_0L)}$, $\Gamma_2 = \frac{1}{j\sin(2k_0L)}$, $\Lambda_1 = \frac{\cos(k_0L)}{j\sin(k_0L)}$, $\Lambda_2 = \frac{\cos(2k_0L)}{j\sin(2k_0L)}$, $G^- = \sum_m \mathrm{e}^{jk_{x_m}(-\frac{d}{2})}\frac{a}{d}\frac{k_0}{\sqrt{k_0^2 - k_{x_m}^2}}\mathrm{sinc}^2(\frac{k_{x_m}}{2}w_1)$, $G^+ = \sum_m \mathrm{e}^{jk_{x_m}(\frac{d}{2})}\frac{a}{d}\frac{k_0}{\sqrt{k_0^2 - k_{x_m}^2}}\mathrm{sinc}^2(\frac{k_{x_m}}{2}w_1)$, $H = \sum_m \frac{a}{d}\frac{k_0}{\sqrt{k_0^2 - k_{x_m}^2}}\mathrm{sinc}^2(\frac{k_{x_m}}{2}w_1)$.

Variables $v_1, v_1', v_2, v_2{}'$can be derived from those equations. The transmission coefficients (T_m) and reflection coefficients (R_m) are calculated as follows:

$$T_m(k_{x_0}) = \frac{a}{d}\frac{k_0}{\sqrt{k_0^2 - k_{x_m}^2}}\mathrm{sinc}\left(\frac{k_{x_m}}{2}w_1\right)[v_1' + 2\mathrm{e}^{jk_{x_m}\left(\frac{d}{2}\right)}v_2']$$

$$R_0(k_{x_0}) = 1 - \frac{a}{d}\frac{k_0}{\sqrt{k_0^2 - k_{x_0}^2}}\mathrm{sinc}\left(\frac{k_{x_0}}{2}w_1\right)[v_1 + 2\mathrm{e}^{jk_{x_0}\left(\frac{d}{2}\right)}v_2]$$

$$R_{m\neq 0}(k_{x_0}) = -\frac{a}{d}\frac{k_0}{\sqrt{k_0^2 - k_{x_m}^2}}\mathrm{sinc}\left(\frac{k_{x_m}}{2}w_1\right)[v_1 + 2\mathrm{e}^{jk_{x_m}\left(\frac{d}{2}\right)}v_2]$$

$T_{\mathrm{g}}^{\mathrm{t}}(k_{\mathrm{e}})$ is defined as $T_{-n}(k_{x_0})$ when $k_{x_0} = k_{\mathrm{e}}k_0$; and $T_{\mathrm{g}}^{\mathrm{r}}(k_{\mathrm{e}})$ is defined as $T_{+n}(k_{x_0})$ when $k_{x_0} = k_{\mathrm{e}}k_0 - nk_{\mathrm{G}}$. In both cases, $k_{\mathrm{e}} \in \left[\frac{nk_{\mathrm{G}}}{k_0} - 1, \frac{nk_{\mathrm{G}}}{k_0} + 1\right]$.

Supplementary Note 3: Theoretical transmission coefficient of the two-grating combination, T_{g}

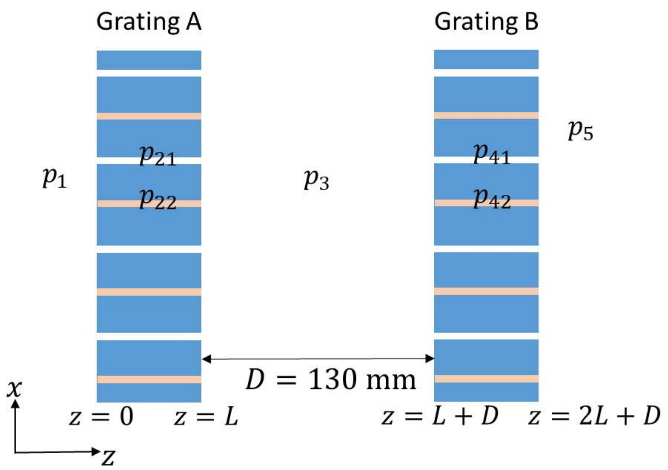

Supplementary Figure 3 Model of two gratings with distance $D = 130$ mm

The pressure field $p_{\mathrm{in}} = \mathrm{e}^{-jk_{x_0}x}$ incidents onto the grating from left. So the pressure fields in areas separated by the boundaries of Grating A and Grating B are:

$p_1 = \mathrm{e}^{j(-k_{x_0}x - k_{z_0}z)} + \sum_m R_m^a \mathrm{e}^{j(-k_{x_m}x + k_{z_m}z)}$, where $k_{x_m} = k_{x_0} + \frac{2\pi}{d}m$, $k_{x_m}^2 + k_{z_m}^2 = k_0^2$,

$p_{21} = A_1 \mathrm{e}^{-jn_1k_0z} + B_1 \mathrm{e}^{jn_1k_0z}$,

$p_{22} = A_2 \mathrm{e}^{-jn_2k_0z} + B_2 \mathrm{e}^{jn_2k_0z}$,

$p_3(z = L) = \mathrm{e}^{j(-k_{x_p}x)}(A_0 + B_0) + \sum_{m \neq p} T_m^a \mathrm{e}^{j(-k_{x_m}x - k_{z_m}(z-L))}$,

$p_3(z = L + D) = \mathrm{e}^{j(-k_{x_p}x)}(A_0 \mathrm{e}^{j(-k_{z_p}D)} + B_0 \mathrm{e}^{j(k_{z_p}D)}) + \sum_{m \neq p} R_m^b \mathrm{e}^{j(-k_{x_m}x + k_{z_m}(z-L-D))}$,
where p is the mode number when $\left|k_{x_p}\right| \leq k_0$,

$p_{41} = A_3 \mathrm{e}^{-jn_1k_0(z-L-D)} + B_3 \mathrm{e}^{jn_1k_0(z-L-D)}$,

$p_{42} = A_4 \mathrm{e}^{-jn_2k_0(z-L-D)} + B_4 \mathrm{e}^{jn_2k_0(z-L-D)}$,

$p_5 = \sum_m T_m^b \mathrm{e}^{j(-k_{x_m}x - k_{z_m}(z-2L-D))}$,

Let $v_1 = A_1 - B_1$, $v_2 = A_2 - B_2$, $v_3 = A_1 \mathrm{e}^{-jn_1k_0L} - B_1 \mathrm{e}^{jn_1k_0L}$, $v_4 = A_2 \mathrm{e}^{-jn_2k_0L} - B_2 \mathrm{e}^{jn_2k_0L}$, $v_5 = A_3 - B_3$, $v_2 = A_4 - B_4$, $v_3 = A_3 \mathrm{e}^{-jn_1k_0L} - B_3 \mathrm{e}^{jn_1k_0L}$, $v_4 = A_4 \mathrm{e}^{-jn_2k_0L} - B_4 \mathrm{e}^{jn_2k_0L}$. By matching the pressure and perpendicular velocity at the four surfaces of the two gratings, we obtain the expressions for $v_1, v_2, v_3, v_4, v_5, v_6, v_7, v_8$ as follows:

$$[H+\Lambda_1]v_1+2G^+v_2-\Gamma_1 v_3=2\mathrm{sinc}\left(\frac{k_{x_0}}{2}w_1\right)$$

$$G^-v_1+[2H+\Lambda_2]v_2-\Gamma_2 v_4=2\mathrm{sinc}\left(\frac{k_{x_0}}{2}w_1\right)\mathrm{e}^{jk_{x_0}(-\frac{d}{2})}$$

$$-\mathit{\Gamma}_1 v_1+\left[H_\mathrm{p}+\mathit{\Lambda}_1+P_1\right]v_3+\left[2G_\mathrm{p}^{+}+2P_1\mathrm{e}^{\frac{jk_{x_p}d}{2}}\right]v_4-P_2v_5-2P_2\mathrm{e}^{\frac{jk_{x_p}d}{2}}v_6=0$$

$$-\Gamma_2 v_2+[G_\mathrm{p}^{-}+P_1\mathrm{e}^{\frac{-jk_{x_p}d}{2}}]v_3+\left[2H_\mathrm{p}+\Lambda_2+2P_1\right]v_4-P_2\mathrm{e}^{\frac{-jk_{x_p}d}{2}}v_5-2P_2v_6=0$$

$$-P_2v_3-2P_2\mathrm{e}^{\frac{jk_{x_p}d}{2}}v_4+\left[H_\mathrm{p}+\mathit{\Lambda}_1+P_1\right]v_1+[2G_\mathrm{p}^{+}+2P_1\mathrm{e}^{\frac{jk_{x_p}d}{2}}]v_2-\Gamma_1 v_7=0$$

$$-P_2\mathrm{e}^{\frac{-jk_{x_p}d}{2}}v_3-2P_2v_4+[G_\mathrm{p}^{-}+P_1\mathrm{e}^{\frac{-jk_{x_p}d}{2}}]v_5+\left[2H_\mathrm{p}+\Lambda_2+2P_1\right]v_6-\Gamma_2 v_8=0$$

$$-\mathit{\Gamma}_1 v_5+[H+\mathit{\Lambda}_1]v_7+2G^+v_8=0$$

$$-\Gamma_2 v_6+G^-v_7+[2H+\Lambda_2]v_8=0$$

In those expressions, $\Gamma_1=\frac{1}{j\sin(k_0L)}$, $\Gamma_2=\frac{1}{j\sin(2k_0L)}$, $\Lambda_1=\frac{\cos(k_0L)}{j\sin(k_0L)}$, $\Lambda_2=\frac{\cos(2k_0L)}{j\sin(2k_0L)}$, $G^-=\sum_m \mathrm{e}^{jk_{x_m}(-\frac{d}{2})}\frac{a}{d}\frac{k_0}{\sqrt{k_0^2-k_{x_m}^2}}\mathrm{sinc}^{\,2}(\frac{k_{x_m}}{2}w_1)$, $G^+=\sum_m \mathrm{e}^{jk_{x_m}(\frac{d}{2})}\frac{a}{d}\frac{k_0}{\sqrt{k_0^2-k_{x_m}^2}}\mathrm{sinc}^{\,2}(\frac{k_{x_m}}{2}w_1)$, $H=\sum_m \frac{a}{d}\frac{k_0}{\sqrt{k_0^2-k_{x_m}^2}}\mathrm{sinc}^{\,2}(\frac{k_{x_m}}{2}w_1)$, $G_\mathrm{p}^{-}=\sum_{m\neq p}\mathrm{e}^{jk_{x_m}(-\frac{d}{2})}\frac{a}{d}\frac{k_0}{\sqrt{k_0^2-k_{x_m}^2}}\mathrm{sinc}^{\,2}(\frac{k_{x_m}}{2}w_1)$, $G_p^{+}=\sum_{m\neq p}e^{jk_{x_m}(\frac{d}{2})}\frac{a}{d}\frac{k_0}{\sqrt{k_0^2-k_{x_m}^2}}\mathrm{sinc}^{\,2}(\frac{k_{x_m}}{2}w_1)$, $H_\mathrm{p}=\sum_{m\neq p}\frac{a}{d}\frac{k_0}{\sqrt{k_0^2-k_{x_m}^2}}\mathrm{sinc}^{\,2}(\frac{k_{x_m}}{2}w_1)$, $P_1=\frac{a}{d}\frac{k_0}{\sqrt{k_0^2-k_{x_p}^2}}\mathrm{sinc}^{\,2}\left(\frac{k_{x_p}}{2}w_1\right)\frac{\cos(k_{z_p}D)}{j\sin\left(k_{z_p}D\right)}$, $P_2=\frac{a}{d}\frac{k_0}{\sqrt{k_0^2-k_{x_p}^2}}\mathrm{sinc}^{\,2}\left(\frac{k_{x_p}}{2}w_1\right)\frac{1}{j\sin\left(k_{z_p}D\right)}$, where $\left|k_{x_p}\right|\leq k_0$.

Variables $v_1,v_2,v_3,v_4,v_5,v_6,v_7,v_8$ can be derived fro those equations. The transmission coefficients (T_m) and reflection coefficients (R_m) are calculated as follows:

$$T_m^\mathrm{b}(k_{x_0})=\frac{a}{d}\frac{k_0}{\sqrt{k_0^2-k_{x_m}^2}}\mathrm{sinc}\left(\frac{k_{x_m}}{2}w_1\right)[v_7+2\mathrm{e}^{jk_{x_m}\left(\frac{d}{2}\right)}v_8]$$

$$R_0^\mathrm{a}(k_{x_0})=1-\frac{a}{d}\frac{k_0}{\sqrt{k_0^2-k_{x_0}^2}}\mathrm{sinc}\left(\frac{k_{x_0}}{2}w_1\right)[v_1+2\mathrm{e}^{jk_{x_0}\left(\frac{d}{2}\right)}v_2]$$

$$R_{m\neq0}^\mathrm{a}(k_{x_0})=-\frac{a}{d}\frac{k_0}{\sqrt{k_0^2-k_{x_m}^2}}\mathrm{sinc}\left(\frac{k_{x_m}}{2}w_1\right)[v_1+2\mathrm{e}^{jk_{x_m}\left(\frac{d}{2}\right)}v_2]$$

$T_\mathrm{g}(k_\mathrm{e})$ is defined as $\mathrm{T}_0^\mathrm{b}(k_{x_0})$ when $k_{x_0}=k_\mathrm{e}k_0$.

Supplementary Note 4: COMSOL simulation setups

The pressure acoustics module in COMSOL Multiphysics 5.1 is used in all the simulations. The background medium is air. The HRs, channels and the waveguides are all modeled as rigid walls (with infinitely large acoustic impedance).

Transmission coefficient of the filter layer

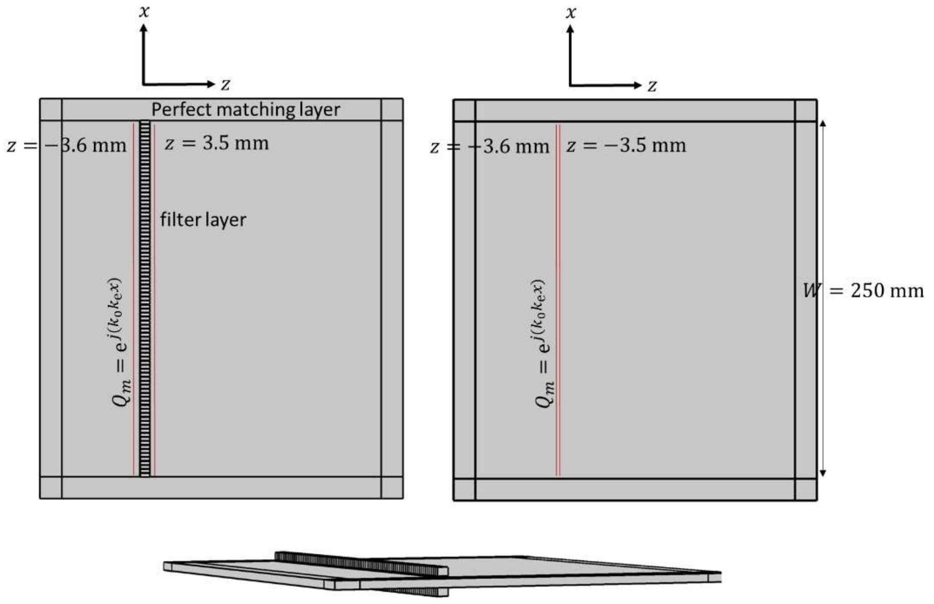

Supplementary Figure 4 Simulation setup for filter layer

The filter layer is composed of two HR arrays. Each array has 90 Helmholtz resonators with period of 2.75 mm, resulting in a structure with size 250 mm in the x direction. A waveguide of height in y direction $h = 5$ mm and width in x direction $W = 250$ mm is inserted between the two HR arrays. The center of the HR arrays is placed at $z = 0$. The waveguide is surrounded by perfectly matched layers. A monopole line source with distribution $Q_m = \exp[i(k_0 k_e x)]$ is set along the x direction at $z = -3.6$ mm. At frequency $f = 9000$Hz, the pressure distribution along x direction at $z = 3.5$ mm is obtained through the frequency domain full wave simulation. For each input $k_e \in [0, 5]$, the spatial Fourier transform of the output pressure distribution is obtained and denoted by $p_{out}(k_e', k_e)$, which is the output pressure with wave vector k_e' when the input effective wave vector is k_e. The same monopole line source with distribution $Q_m = \exp[i(k_0 k_e x)]$ is set along the x direction at $z = -3.6$ mm of an empty waveguide and the spatial Fourier transform of pressure distribution at $z = -3.5$ mm is obtained as $p_{ref}(k_e', k_e)$. The transmission coefficient of the filter layer, T_f, is defined as $T_f(k_e) = \frac{p_{out}(k_e, k_e)}{p_{ref}(k_e, k_e)}$.

Transmission coefficient of the grating layer

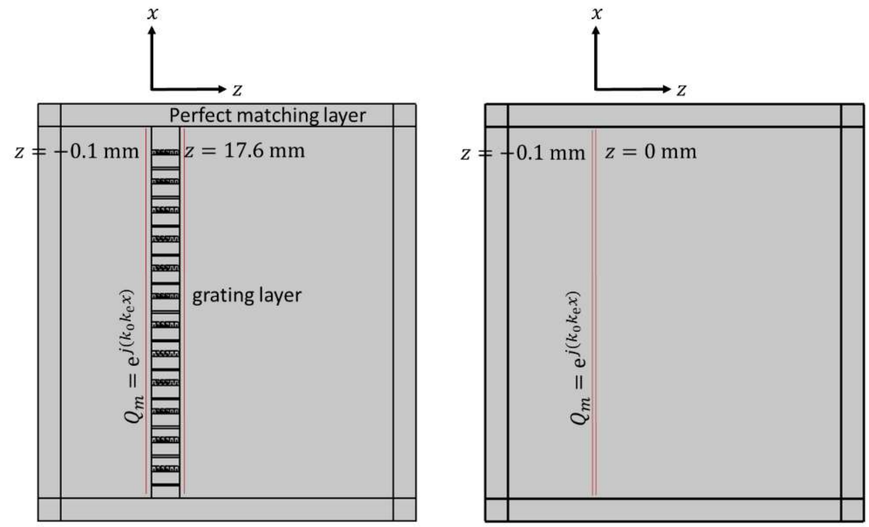

Supplementary Figure 5 Simulation setup for single grating

The grating layer is 250 mm in x direction, which is designed to have the same size as the filter layer. For three different grating periods $d_2 = 18.9\text{ mm}, 12.6\text{ mm}, \text{and } 9.4\text{ mm}$, the numbers of π phase shifter and 2π phase shifter pairs are 13, 20, and 26, respectively. The grating layer is put into the same waveguide that is used for the simulation of filter layers. The entrance side of the grating is set at $z = 0$ mm. A monopole line source with distribution $Q_m = \exp[i(k_0 k_\mathrm{e} x)]$ is set along the x direction at $z = -0.1$ mm. At frequency $f = 9000$ Hz, the pressure distribution along x direction at $z = 17.6\text{ mm}$ (0.1 mm after the exit of the grating) is obtained through the frequency domain full wave simulation. For each input k_e, the spatial Fourier transform of the output pressure distribution is $p_\mathrm{out}(k_\mathrm{e}', k_\mathrm{e})$, which is the output pressure with wave vector k_e' when the input effective wave vector is k_e. The same monopole line source with distribution $Q_m = \exp[i(k_0 k_\mathrm{e} x)]$ is set along the x direction at $z = -0.1\text{ mm}$ of an empty waveguide and the spatial fourier transform of pressure distribution at $z = 0\text{ mm}$ is $p_\mathrm{ref}(k_\mathrm{e}', k_\mathrm{e})$. The transmission coefficients of the grating layer, T_g^t and T_g^r, are calculated as $T_\mathrm{g}^\mathrm{t}(k_\mathrm{e}) = \frac{p_\mathrm{out}(k_\mathrm{e}-k_\mathrm{G}, k_\mathrm{e})}{p_\mathrm{ref}(k_\mathrm{e}, k_\mathrm{e})}$, $T_\mathrm{g}^\mathrm{r}(k_\mathrm{e}) = \frac{p_\mathrm{out}(k_\mathrm{e}, k_\mathrm{e}-k_\mathrm{G})}{p_\mathrm{ref}(k_\mathrm{e}-k_\mathrm{G}, k_\mathrm{e}-k_\mathrm{G})}$, where $k_\mathrm{e} \in \left[\frac{k_\mathrm{G}}{k_0} - 1, \frac{k_\mathrm{G}}{k_0} + 1\right]$.

Image transfer function of the whole structure

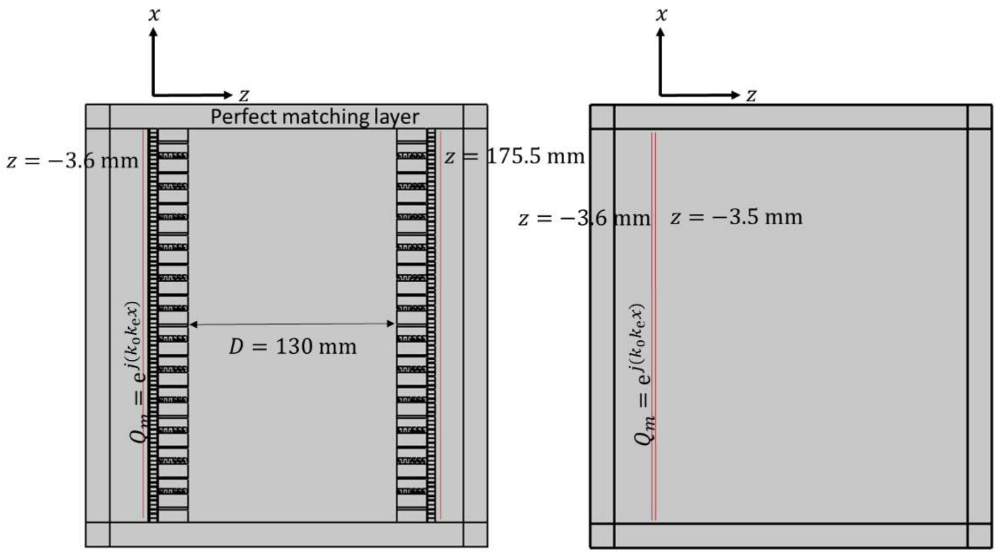

Supplementary Figure 6 Simulation setup for the whole system

In the simulation, the transmitter and the receiver are considered as a whole system. They are placed in the same waveguide as previous simulations. The input plane is at $z = -3.6$ mm. The entrance of the grating layer in the transmitter is at $z = 3.5$ mm. The distance between the two gratings is 130 mm. And the output plane is at $z = 178$ mm. A monopole line source with distribution $Q_m = \exp[i(k_0 k_\mathrm{e} x)]$ is set along the x direction at $z = -3.6$ mm. At frequency $f =$ 9000 Hz, the output pressure distribution along *x* direction at $z = 178$ mm is obtained through the frequency domain full wave simulation. For each input $k_\mathrm{e} \in [0, 5]$, the spatial Fourier transform of the output pressure distribution is $p_\mathrm{out}(k'_\mathrm{e}, k_\mathrm{e})$, which is the output pressure with wave vector k'_e when the input effective wave vector is k_e. The same monopole line source with distribution $Q_m = \exp[i(k_0 k_\mathrm{e} x)]$ is set along the x direction at $z = -3.6$ mm of an empty waveguide and the spatial Fourier transform of pressure distribution at $z = -3.5$ mm is obtained as $p_\mathrm{ref}(k'_\mathrm{e}, k_\mathrm{e})$. The image transfer function of the whole system, T, is defined as $T(k_\mathrm{e}) = \frac{p_\mathrm{out}(k_\mathrm{e}, k_\mathrm{e})}{p_\mathrm{ref}(k_\mathrm{e}, k_\mathrm{e})}$.

Simulation of edge detection

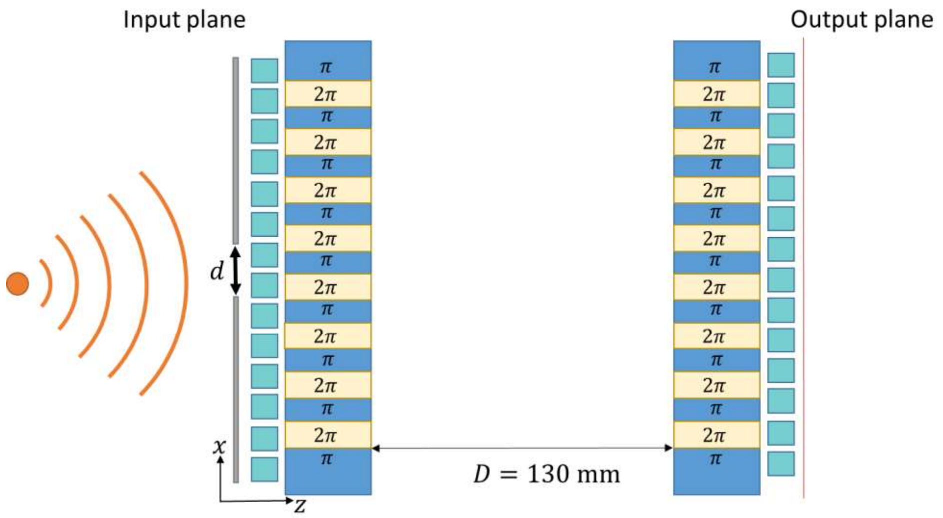

Supplementary Figure 7 Simulation setup for the edge detection

Frequency domain COMSOL simulations are performed for the imaging process of the three devices and the empty waveguide. The slit with width d is put at the input plane. A point monopole source is put at 20 cm away from the slit. The simulation frequency is set at $f = 9000$ Hz. The spatial Fourier transform of the field along the transverse direction (x direction) at the output plane are obtained as $F_n(k_\mathrm{e}), n = 0, 1, 2, 3$. $F_n(k_\mathrm{e})$ is normalized with $B_n = \frac{\max|F_n(k_e)|}{\max|S_\mathrm{r}(k_e)|} \mathrm{e}^{i\phi'_n(k_\mathrm{e})}$, where $\frac{\max|F_n(k_\mathrm{e})|}{\max|S_\mathrm{r}(k_\mathrm{e})|}$ is the ratio of the maximum amplitudes of $F_n(k_\mathrm{e})$ to $S_r(k_\mathrm{e})$ in the range $k_\mathrm{e} \in [n-1, n+1], n = 1,2,3$. The simulation is implemented in the frequency domain. The multiple reflection between the two gratings is included in the frequency response. Thus $\phi_n(k_\mathrm{e})$ is the phase of transmission coefficient $T_\mathrm{continuous} = T_\mathrm{f} T_\mathrm{g} T_\mathrm{f}$ theoretically calculated for lens1, lens2, and lens3. $\phi_0(k_\mathrm{e})$ is the phase delay for wave propagation in distance $D + 2L + 2a_1$, as in the experimental case. The inverse Fourier transforms of the normalized $\frac{F_n(k_\mathrm{e})}{B_n}$ are denoted by $I_n^\mathrm{S}(x)$. The full image is obtained as $I_\mathrm{full}^\mathrm{S}(x) = \sum_{n=0}^{3} I_n^\mathrm{S}(x)$. The edge image is obtained as $I_\mathrm{edge}^\mathrm{S}(x) = \sum_{n=1}^{3} I_n^\mathrm{S}(x)$.

Supplementary Reference

[1] Nicholas Fang, Dongjuan Xi, Jianyi Xu, Muralidhar Ambati, Werayut Srituravanich, Cheng Sun, and Xiang Zhang, "Ultrasonic metamaterials with negative modulus," Nature materials 5, 452 (2006).

[2] Zixian Liang and Jensen Li, "Extreme acoustic metamaterial by coiling up space," Physical review letters 108, 114301 (2012).

[3] Johan Christensen, Luis Martin-Moreno, and Francisco Jose Garcia-Vidal, “Theory of resonant acoustic transmission through subwavelength apertures," Physical review letters 101, 014301 (2008).
